# An Improved Chamber for Direct Visualisation of Chemotaxis

**DOI:** 10.1371/journal.pone.0015309

**Published:** 2010-12-14

**Authors:** Andrew J. Muinonen-Martin, Douwe M. Veltman, Gabriela Kalna, Robert H. Insall

**Affiliations:** CRUK Beatson Institute for Cancer Research, Glasgow, United Kingdom; University of Bergen, Norway

## Abstract

There has been a growing appreciation over the last decade that chemotaxis plays an important role in cancer migration, invasion and metastasis. Research into the field of cancer cell chemotaxis is still in its infancy and traditional investigative tools have been developed with other cell types and purposes in mind. Direct visualisation chambers are considered the gold standard for investigating the behaviour of cells migrating in a chemotactic gradient. We therefore drew up a list of key attributes that a chemotaxis chamber should have for investigating cancer cell chemotaxis. These include (1) compatibility with thin cover slips for optimal optical properties and to allow use of high numerical aperture (NA) oil immersion objectives; (2) gradients that are relatively stable for at least 24 hours due to the slow migration of cancer cells; (3) gradients of different steepnesses in a single experiment, with defined, consistent directions to avoid the need for complicated analysis; and (4) simple handling and disposability for use with medical samples. Here we describe and characterise the Insall chamber, a novel direct visualisation chamber. We use it to show GFP-lifeact transfected MV3 melanoma cells chemotaxing using a 60x high NA oil immersion objective, which cannot usually be done with other chemotaxis chambers. Linear gradients gave very efficient chemotaxis, contradicting earlier results suggesting that only polynomial gradients were effective. In conclusion, the chamber satisfies our design criteria, most importantly allowing high NA oil immersion microscopy to track chemotaxing cancer cells in detail over 24 hours.

## Introduction

Cell motility is one of the defining characteristics of invasive tumours and involves a series of co-ordinated processes requiring cell protrusion, adhesion, contraction and de-adhesion for the cell to move forward [1], [2]. Chemotaxis is the process by which the direction of motile cells is biased along a concentration gradient of soluble factors/extracellular signals [3]. This is distinct from chemokinesis, the random migration of cells observed in a homogenous solution of an extracellular signal [2]. This evolutionarily ancient behaviour can be seen across species, from amoebas to eukaryotic cells [4], [5]. Chemotaxis is a key feature of cell motility, and there has been a growing appreciation over the last decade that it plays an important role in cancer cell migration, invasion and metastasis [6], [7]. Many of the proteins that regulate motility and chemotaxis are also markers for metastasis and poor patient outcomes [8].

Cell migration involving chemotaxis requires a complex set of interacting processes that includes detection of the attractant, extraction and integration of information about the source's direction and transmission of the information to the cell's motility machinery. Significant understanding about chemotaxis has been derived from research on the social amoeba Dictyostelium discoideum. This experimentally friendly tool has allowed the dissection and greater appreciation of multiple, intertwined signalling pathways [9]. The challenge now is to use this knowledge to enhance our understanding of the role of chemotaxis in human tumours. Drugs which target cancer cell invasion and metastasis have proven difficult to develop, so an improved understanding of the detailed mechanism of chemotaxis may enable the prioritisation of better drug targets, better drugs, appropriate patient selection and smarter early clinical study design [10].

Research into the field of cancer chemotaxis is still in its infancy and investigative tools have had to be developed and refined, as many were initially designed for investigating rapidly moving cells like neutrophils and Dictyostelium. Most assays use the two-well design whereby cells are seeded between the wells, one containing a control or buffer substance and the other the chemoattractant. The cells lying within the gradient are free to migrate between them. These assays can be divided into direct and indirect visualisation assays with various advantages, disadvantages and caveats.

The choice of assay depends on the research question being asked. Indirect assays are generally useful for screening chemoattractants and rapidly performing multiple simultaneous experiments. The most commonly employed indirect assay is the Boyden/Transwell assay. Quantitative data is gained by counting cells that have migrated to the chemoattractant well in a fixed time and by using checkerboard analysis the relative effects of chemokinesis and chemotaxis can then be calculated. But therein lies the fundamental problem with indirect methods, which are only capable of estimating the role of chemotaxis [11]. Accurate analysis of chemotaxis is further hampered by an unknown concentration gradient over time.

Direct visualisation chambers allow cells to be observed migrating using time-lapse microscopy in real-time and are considered the gold standard assay for investigating chemotaxis [2]. They are capable of accurately quantifying chemotaxis and importantly, are able to distinguish this from chemokinesis as well as providing detailed information about the behaviour of individual cells. Direct visualisation chambers have evolved over the years in response to the need to investigate different cell types under a set of specified conditions. For brevity, we discuss bridge chambers, although others exist including the under agarose [12], pipette [13] and elaborate microfluidic chamber assays [14].

Bridge chambers provide a visualisation platform for observing the behaviour of cells between the two wells. The cells are plated onto cover slips, which are then inverted leaving a small gap between the bridge and the cover slip, too small for fluid flow to occur, but large enough to allow diffusion of the chemoattractant. Cells can then be observed using an inverted time-lapse microscope. This means that individual chemotactic parameters can be recorded as well as separating the steps of the motility cycle, for example lamellipod protrusion and detachment of the rear aspect of the cell [15], [16].

**Figure 1:**
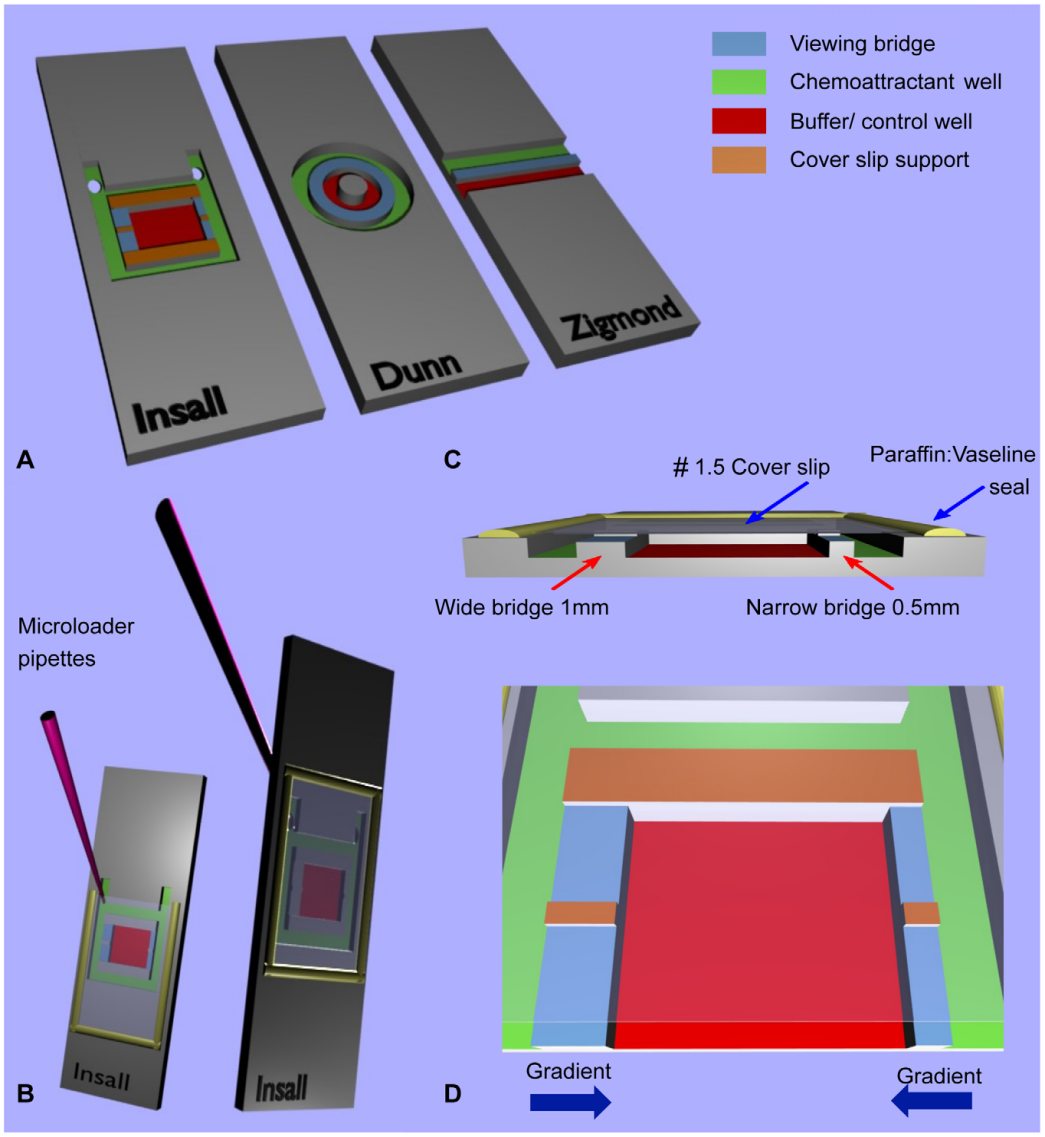
Comparison of bridge chamber features. (A) Schematic showing the Insall, Dunn and Zigmond chambers. The chemoattractant and buffer/control wells have been colour coded for direct comparison, along with the viewing bridges and cover slip supports. Note that the central cylindrical block on the Dunn chamber is the same height as the bridge and therefore offers no support to the cover slip. Fig 1 (B) demonstrates the versatility of the chamber with front or reverse side chemoattractant loading with no requirement for metal clips, unlike the Zigmond chamber. The latter technique involves loading after the cover slip has been secured and sealed in place with a 1∶1 mix of vaseline: paraffin, producing a tight seal that reduces the risk of evaporation during experiments over several hours. Fig 1 (C) Cross section through the Insall chamber highlighting one key feature – the ability to use thin (#1.5, 0.16–0.18 mm) cover slips that permit high NA oil immersion microscopy. Bridges of differing widths provide different gradient steepnesses. Fig (D) provides a close-up of the Insall chamber and demonstrates the directions of the two chemotactic gradients, which are unidirectional across each bridge.

Commercially available alternative chemotaxis bridge assays include the Zigmond and Dunn chambers. The Zigmond chamber (Fig. 1A) was first described in 1977 and was designed for studying polymorphonuclear (PMN) leukocytes capable of rapidly migrating at speeds of up to 30 µm/min. This chamber permits the generation of shallow gradients, allowing the demonstration that these cells could respond to only 1% changes in concentration across their length [17]. The Zigmond chamber was a great improvement on under agarose assays, due to its improved optical properties and near steady state linear gradient stable for 30–90 minutes – perfectly adequate for assessing the rapidly migrating PMN leukocytes. One major flaw with this chamber design when considering its use for investigating cancer cells is the variable gap between the bridge and cover slip. This arises as a result of the cover slip being held in place by temperature sensitive springs, which are capable of deforming the plexiglass chamber, leading to unpredictable gradient variability both during and between experiments [18].

The Dunn chamber (Fig. 1A) was published in 1991 for the investigation of chemotaxis in fibroblasts, which migrate much more slowly at 0.42–1.25 µm/min, similar to the migratory speeds of cancer cells that move around 1 µm/min [19], [20]. The chamber design circumvented the problem of a variable gap between bridge and cover slip by seating a thick (#3, 0.25–0.35 mm thickness) cover slip over a relatively inflexible glass chamber with annular wells of precise geometry and therefore less prone to flexure. Gradient characterisation experiments for this chamber found it was able to form a linear gradient within one hour of setting up the chamber, with a gradient half-life of 10 to 30 hours, due to the stable gap between the cover slip and bridge of 20 µm. The reliable prediction and long-term stability of this chamber make it suitable for investigating the considerably slower fibroblasts. These attributes and in particular a similar gap were considered important for our new chamber, but there are limitations when using this assay for cancer cell chemotaxis. Firstly, data analysis is complicated by the annular bridge design resulting in variable chemoattractant gradient orientation. Secondly, the use of relatively thick #3 cover slips precludes the use of high numerical aperture (NA) oil immersion objectives that are generally designed with fixed compensation for thinner (#1.5, 0.16–0.18 mm thickness) cover slips. This has meant that the critical work investigating the mechanisms of chemotaxis with fluorescently tagged proteins in known gradients has been very technically challenging. In addition, changing the gradient in Dunn chambers is clumsy and inefficient, contributing significantly to the number of experiments that are unsuccessful.

Here we describe an improved direct visualisation chamber and its use for measuring melanoma chemotaxis. The Insall chamber is a refined derivative of the Dunn chemotaxis chamber. Particular advantages include easy handling, gradients with defined directions and two different gradient steepnesses in the same assay, and above all the ability to use normal thin (#1.5) cover slips, allowing the use of high NA oil immersion lenses. As with the Dunn chamber, gradients are maintained for at least 24 hours, allowing slowly-moving cancer cells to be tracked.

## Results and Discussion

### Fabrication and Geometry

**Figure 2:**
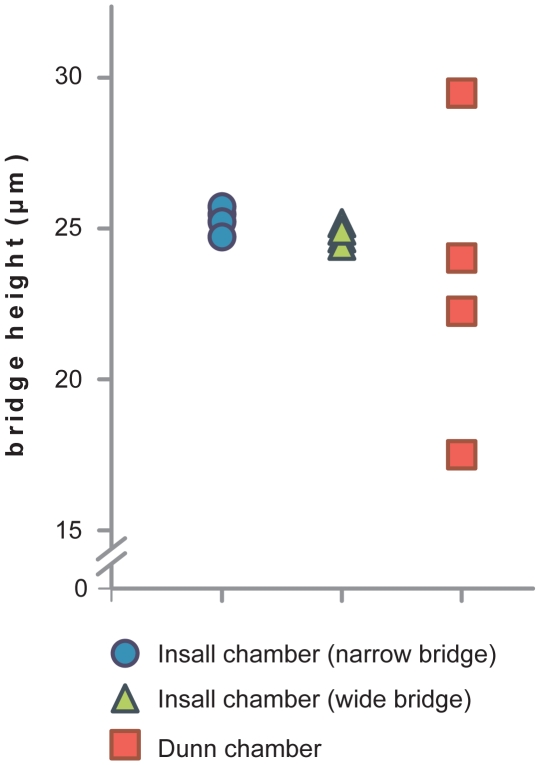
Bridge heights in Insall and Dunn chambers during use. Bridge heights are measured in µm at 4 locations across the Insall chamber narrow and wide bridges and the Dunn chamber using a #1.5, 0.16–0.18 mm cover slip. Chambers were filled with fluorescein and images were taken in a z-stack using 1 µm intervals to calculate the distance between the bridge and cover slip. The bridge height was consistent at all sites in the Insall chamber with less than 1 µm variation demonstrating that thin cover slips can be used without affecting the geometry of the chamber and hence the diffusion profile.

Chambers were microfabricated by Epigem Ltd. (Redcar, UK) from two-layered, optically clear and nonpolarising polymethyl methacrylate (PMMA) blanks coated with a 20 µm layer of SU8 photoresist. The supports at the edge of the chamber and the centre of the slide were crosslinked from the SU8 using UV light, then following development mechanical milling was used to remove 1 mm of material to make the wells. To allow the use of thin cover slips – impossible in Dunn and Zigmond chambers because the cover slips bend and occlude the chamber – we included supports within the bridges (Fig. 1A,D & Fig. 2), at the same height as the edges of the chamber. These minimise the length over which the cover slip must span. Furthermore, the cover slip can easily be sealed in place avoiding the need for cover slip springs, unlike the Zigmond chamber, which can potentially deform the chamber and distort the gradient.

### Gradients with consistent directions

Since the chamber was microfabricated, rather than ground from glass as is the case for Dunn chambers, it was possible to make the bridge area square, giving a consistent geometry for the bridges and direction for the gradients that were formed (Fig. 1D). This makes analysis of the directions of cells easier than for Dunn chambers [21]. We also included additional supports for the cover slip at the top and bottom of the chamber, which increases the stability of the gradient by limiting the amount of diffusion as well as providing support.

### Gradients with different steepnesses

We were also able to create different bridge widths in the two sides of the device (Fig. 1C,D). Since the concentrations of attractant at the outside and inside wells are constant, this translates into two different steepnesses of gradients. With an automated XY stage it is thus possible to track the chemotaxis in both gradients simultaneously. In the version of the chamber shown here we used bridge widths of 0.5 mm and 1.0 mm; we have successfully used other sizes, up to 2.5 mm, though the gradients form relatively slowly in longer bridges.

### Gradient stability and linearity

**Figure 3:**
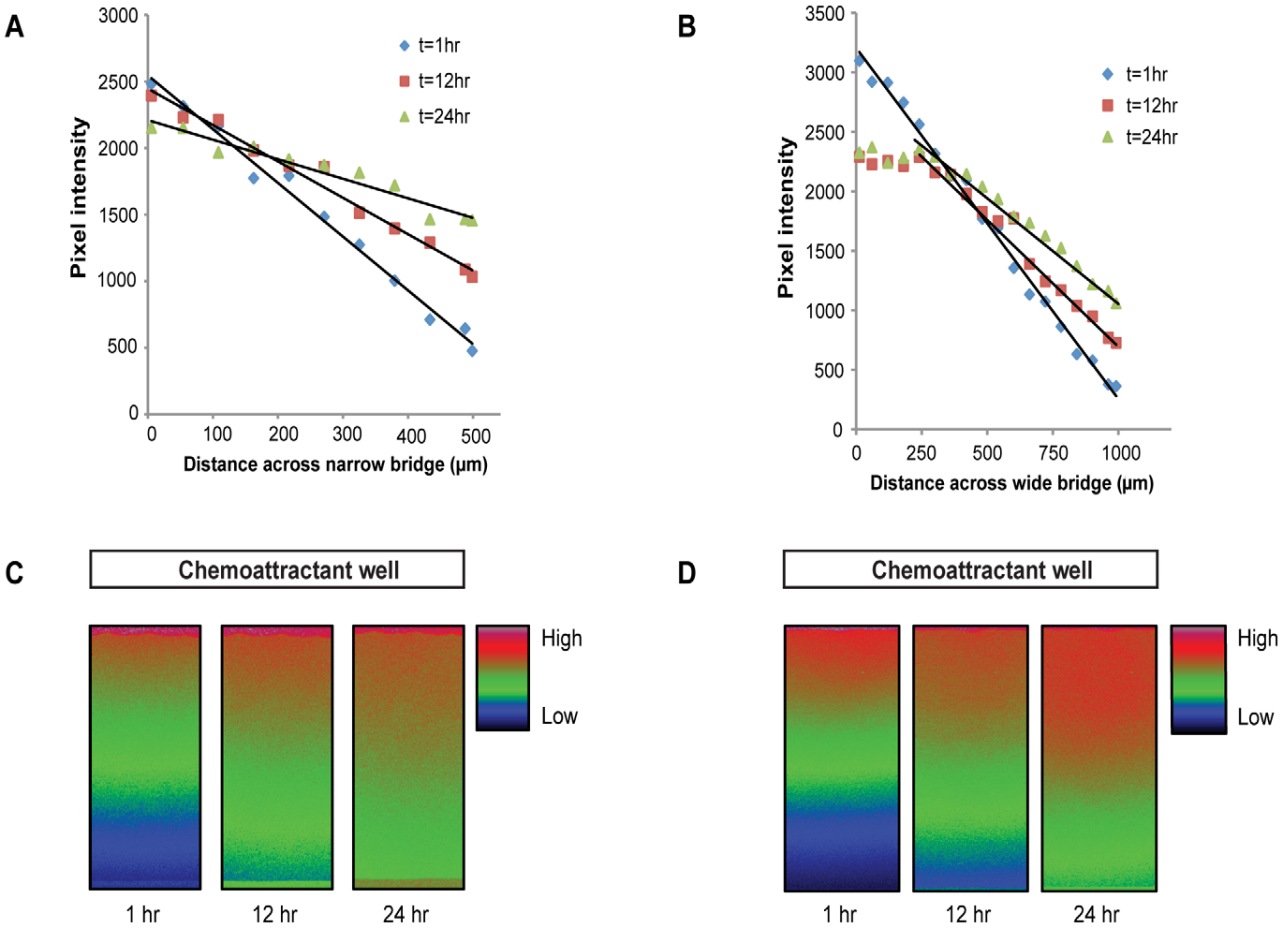
Stability of linear gradients. Gradients were established for between 1 and 24 hours across the (A) narrow and (B) wide bridges in the Insall chamber. The outer chemoattractant chamber was loaded with 100 µM fluorescein in 100 mM Tris·Cl pH 8.0 and the inner control chamber with 100 mM Tris·Cl pH 8.0. Confocal images were acquired at various intervals and quantification of the gradient was performed by measuring pixel intensity from the fluoresence signal as it diffused across the (C) narrow and (D) wide bridges.

Results from tests using fluorescein demonstrated that the profiles of both gradients remain stable for 24 hours (Fig. 3). The narrow (0.5 mm width) bridge produces a steep linear gradient and the wide (1.0 mm) bridge a less steep gradient, but both are stable through 24 hours. With time, the steepness of both gradients gently reduced slightly, but chemotaxis was still observed. Interestingly, the wider (1 mm width) bridge produced a stable linear gradient again with gentle reduction in steepness, but only over the two-thirds of the bridge nearest the buffer following initial stabilisation. We therefore conclude that the central area of the wide bridge should be imaged for consistency. This conflicts with Saadi et al [22], who investigated chemotaxis of MDA-MB-231 cells in their parallel-gradient microfluidic chamber in response to linear and polynomial EGF gradient profiles. Their data suggested linear gradients were not effective in inducing chemotaxis whereas polynomial gradients did induce a chemotactic response. Our data suggest that two different linear gradient profiles of serum can induce chemotaxis in MV3 melanoma cells. The effect of differing gradient profiles on cancer cell chemotaxis is therefore clearly an area that merits further research. Our data justify the use of a relatively long (8–24 hour) assay period because the relatively short 3 hour assay period used by Saadi et al and for example the transwell assays may produce conflicting results due to the refractory window during which locomotion can be reduced to below the haptokinetic baseline [23].

### Ease of Use

With a similar principle and methodology, the chamber is as easy to handle as the Dunn chamber, but with a few advantages. The “rabbit ears” at the top of the external well (Fig. 1C) facilitate the filling and refilling of the external well. These can if desired be drilled to allow the contents of the chemoattractant/outer well to be changed in situ by reverse filling (Fig. 1B). We prefer this latter option for investigating slow moving cancer cells over longer time frames because in our experience, the chamber can be sealed more effectively, which reduces the risk of evaporation and also the risk of shearing cells against support structures whilst setting up the chamber (Fig. 1B). In the future it will also be possible to automate the replacement of buffer in the outer chamber, with obvious improvements in the possible range of experiments. Finally, the chambers are considered disposable with relatively cheap manufacturing costs and so are suitable for the investigation of chemotaxis using fresh human cancer cell samples.

### Microscopy

**Figure 4:**
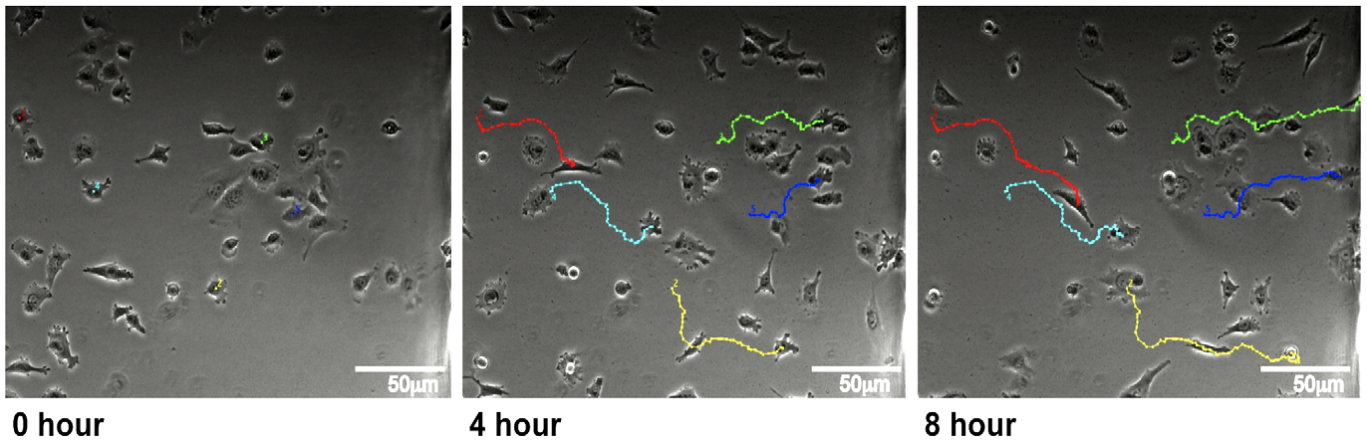
Insall chamber chemotaxis assay. Figure shows chemotaxis of MV3 melanoma cells towards a 10% FBS gradient on the right of each image and imaged using inverted phase contrast microscopy with a 10x phase objective with time-lapse images taken every 5 minutes for 8 hours. Cell tracking lines have been created using the ImageJ MTrackJ plugin.

**Figure 5:**
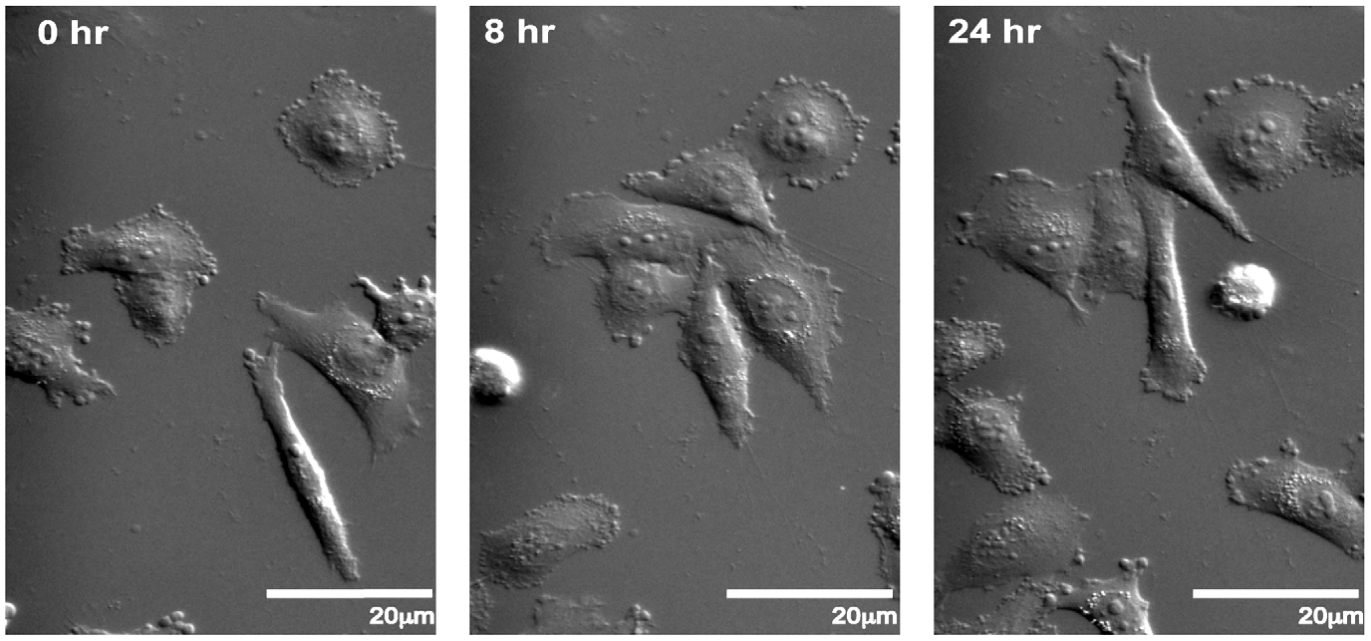
DIC imaging of melanoma chemotaxis. MV3 melanoma cells migrating towards a 10% FBS chemoattractant at the top of the image(s) over the course of 24 hours were imaged in differential image contrast using a 40x 1.3NA objective.

**Figure 6:**
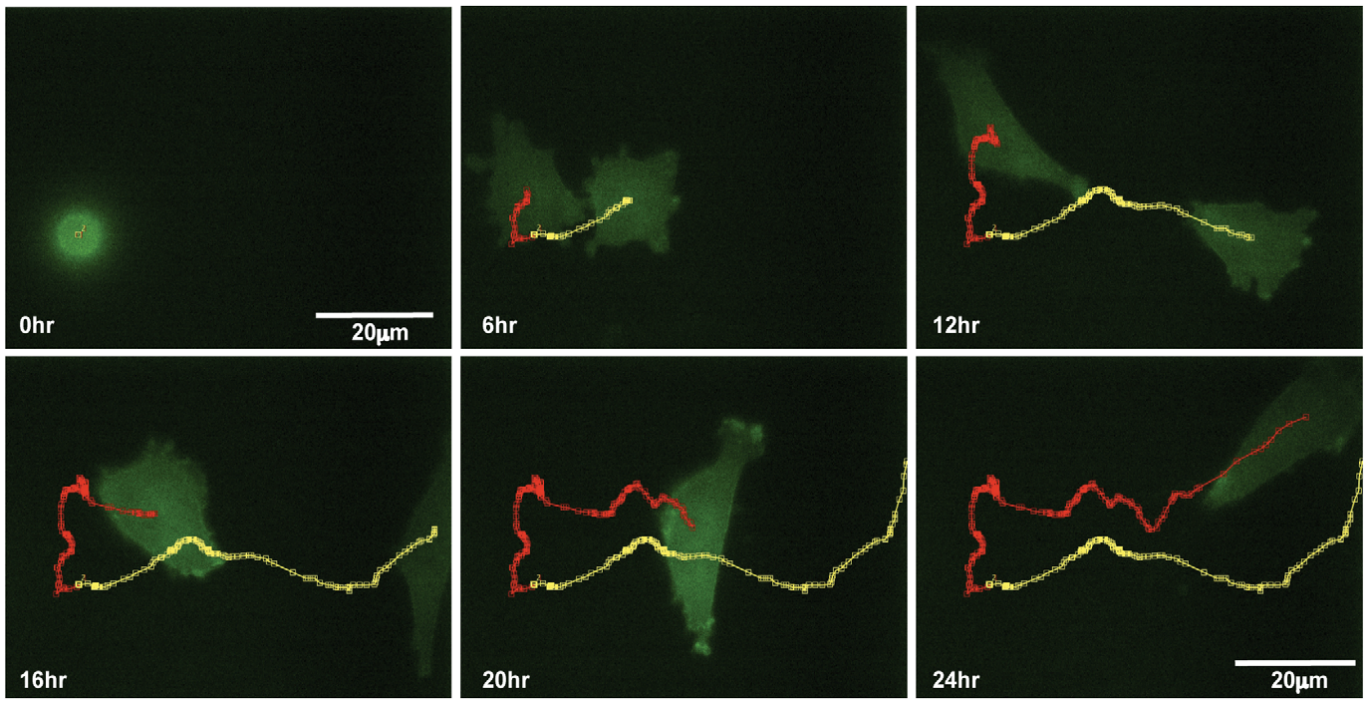
Live cell fluorescence microscopy using high-NA objective. Insall chamber chemotaxis assay with identical set-up and analysis to experiment in Fig. 3, but the MV3 cells are stably transfected with GFP-Lifeact. Time-lapse images are taken every 5 minutes for 24 hours with and imaged with a 60x, 1.4NA oil immersion objective. Two daughter cells can be seen emerging from mitosis, polarising and then chemotaxing towards the FBS gradient. Their paths are highlighted by the red and yellow tracking lines.

Using time-lapse phase contrast microscopy with an inverted microscope we have been able to successfully demonstrate the chemotaxis of MV3 melanoma cells towards a 10% Foetal Bovine Serum (FBS) gradient, with cell paths tracked using the ImageJ MTrackJ plugin (Fig. 4). This can be successfully and reliably performed using a range of objectives from 10x to 60x magnification. The use of nonpolarising substrates in the chamber fabrication means that differential interference contrast (DIC) microscopy may also be used, which is not possible with many plastic devices. This technique provides high quality images and detail including intracellular organelles can clearly be seen (Fig. 5). Due to the thin cover slips high NA oil immersion microscopy is also possible and we demonstrate the chemotaxis of Lifeact-GFP transfected MV3 cells over 24 hours using fluorescence microscopy with a 60x 1.45NA oil immersion objective (Fig. 6).

### Quantification and statistical analysis

In order to quantify chemotaxis with MV3 cells, we analysed the cell tracks of 43 cells in the presence of a chemoattractant (SFM inner well: FBS outer well – denoted as SFM:FBS) and 46 cells without a chemoattractant (SFM inner well: SFM outer well – denoted as SFM:SFM) migrating on the narrow bridge. Data was collected over 12 hours from both experiments running simultaneously in the same microscope incubator (Movie S1 and Movie S2) and in the chemoattractant experiment the chemoattractant is to the right.

**Figure 7:**
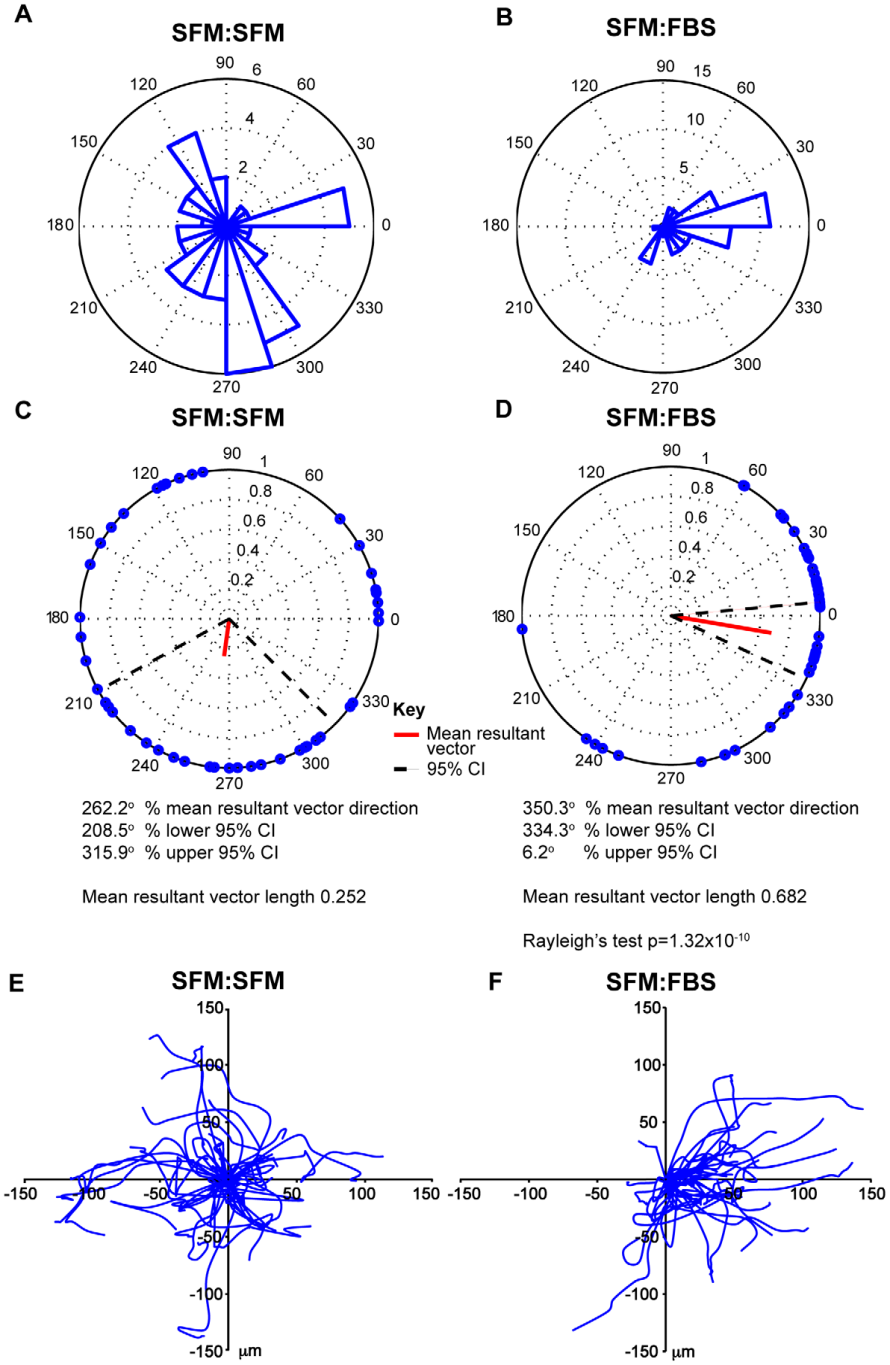
Quantification and statistical analysis. (A, C, E) represent the results for the migration of all 46 MV3 cells tracked in a control experiment (SFM:SFM) and (B, D, F) represent the results for the migration of all 43 MV3 cells in the presence of a chemoattractant (SFM:FBS) both across the narrow bridge over 12 hours. In the chemoattractant experiment the chemoattractant is to the right. Rose plots (A & B) demonstrate that cells migrated in nearly all directions in the control experiment but predominantly up the gradient in the chemoattractant experiment. The polar plots for the control experiment (C) reveal a short mean resultant vector for the control experiment with a wide 95% confidence interval not towards the gradient. Conversely, the polar plots for the chemoattractant experiment (D) demonstrate a much longer mean resultant vector with corresponding narrow 95% confidence intervals in the direction of the chemoattractant. The evidence for directed migration is further supported by a highly significant Rayleigh test (p = 1.32×10^−10^) demonstrating a unimodal deviation from uniformity or random migration in this instance. The paths of individual cells in the spider plots highlight a strong bias for migration towards the chemoattractant.

The quantification and analysis of the data demonstrating chemotaxis in the presence of 10% FBS and random migration with no chemotactic gradient can be seen in (Fig. 7). Rose plots (A & B) demonstrate that cells migrated in nearly all directions in the control experiment but predominantly up the gradient in the chemoattractant experiment. Polar plots use end-point data to plot points on a unit circle and the red line indicates the direction and magnitude of the resultant mean vector [24]. The polar plots for the control experiment (C) reveal a short mean resultant vector for the control experiment with a wide 95% confidence interval not towards the gradient. Conversely, the polar plots for the chemoattractant experiment (D) demonstrate a much longer mean resultant vector with corresponding narrow 95% confidence intervals in the direction of the chemoattractant. The evidence for directed migration is further supported by a highly significant Rayleigh test (p = 1.32×10^−10^) demonstrating a unimodal deviation from uniformity or random migration in this instance. The paths of individual cells in the spider plots highlight a strong bias for migration towards the chemoattractant.

### Conclusion

In conclusion, we have created a novel chemotaxis chamber for studying cancer cell chemotaxis. The Insall chamber satisfies our design criteria and, most importantly, it allows high numerical aperture oil immersion microscopy to track and investigate cancer cell chemotaxis for at least 24 hours.

## Materials and Methods

### Chemotaxis chamber fabrication

The Insall chamber (Fig. 1) is manufactured from polymethyl methacrylate (PMMA) by Epigem Ltd as described in the Results & Discussion section. Where appropriate, two 1 mm holes were drilled in house right through the distal legs of the chamber at 45° away from the well. This two-well bridge design assay consists of a square closed central chamber separated from the square outer chamber by two bridges on opposite sides designed to lie 20–30 µm below the surface of the cover slip once in situ. In order to use a thin #1.5 cover slip, supports have been built into the design both in the centre of and around the bridges. The bridges both measure 3 mm in length, with the widths of the narrow and wide bridges measuring 0.5 mm and 1 mm respectively (Fig. 1C) producing linear gradients with two different steepnesses.

### Culture of MV3 and Lifeact transfect MV3 melanoma cells

Highly invasive and metastatic MV3 human melanoma cells [25] were cultured in Dulbecco's Modified Eagle's Medium (Invitrogen), supplemented with 2 mM glutamine and 10% Foetal Bovine Serum (FBS; Harlan). MV3 cells transfected with GFP-Lifeact by nucleofection were cultured in the same medium supplemented with G-418S (Formedium Ltd). All cell cultures were stored at 37°C and 5% CO2 in a humidified atmosphere.

### Cover slip preparation

22 mmx22 mm (#1.5, 0.16–0.18 mm) cover slips were acid washed for 15 minutes in 0.1 M HCL before washing with water for a further 15 minutes. The cover slips were then sterilised by washing in 70% ethanol for 15 minutes before drying at room temperature in a laminar flow hood. A 20 mcg/ml fibronectin coating (BD Biosciences) was applied to the sterile cover slips in a 6-well dish and left to adsorb for 1 hour at room temperature. The fibronectin was aspirated and the cover slips washed 3 times in distilled water. 1% BSA (Sigma-Aldrich) to inhibit non-specific adhesion was heat-inactivated for 13 minutes at 85°C and applied to the cover slips for 1 hour at room temperature before being aspirated and the cover slips rinsed 3 times in PBS. MV3 or Lifeact transfected MV3 cells in 2 ml medium were seeded at a density of 4×10^4^ cells/ml and left to spread overnight at 37°C and 5% CO2 in a humidified atmosphere. The medium was aspirated and the cells starved in the MV3 medium described above with 0.1% FBS for 4 hours prior to assembling the chamber.

### Insall chamber assay setup

The inner well is filled with 12.5 µl control medium consisting of MV3 medium without FBS. The prepared cover slip is then inverted and carefully lowered onto the chamber resting one edge before lowering gently to ensure the central chamber remains bubble free. Excess medium is blotted from the edges of the cover slip with care to avoid moving the cover slip, which could result in shearing of the cells over the bridge. The cover slip is then sealed into place with 1∶1 Vaseline:Paraffin (Melting point 58–62°C, Sigma-Aldrich). The outer surface of the cover slip is then washed with distilled water and dried before wiping with lens cleaning tissue. The chamber is then inverted to fill the outer chemoattractant well with approximately 80 µl MV3 medium using a 20 µl microloading tip (Eppendorf). FBS is therefore used as the chemoattractant as in other studies [26]. Holding the Insall chamber at 45 degrees whilst filling allows any bubbles to escape through the drilled holes. The two holes are then sealed with a thin strip of water resistant tape to avoid evaporation. The chemotactic responses of cells overlying the bridge region can then be analysed after incubating at 37°C for one hour to allow the gradient to form.

### Gradient characterisation

The concentration gradient profile was characterised using 100 µM fluorescein isothiocyanate in 100 mM Tris·Cl pH 8.0 in the chemoattractant well and 100 mM Tris·Cl pH 8.0 in the inner control well. Images were captured using a Leica confocal microscope. 16 bit greyscale images were acquired at intervals during 24 hours and pixel intensity across the bridge was quantified using ImageJ software.

### Time-lapse microscopy

A Nikon TE2000-E inverted time-lapse microscope equipped with a 37°C temperature controlled incubation chamber, motorised stage (Prior) and the Perfect Focus System (Nikon). The Insall chambers were mounted cover slip side down on a slide holder and overlapping images across the entire width of both bridges were captured for up to x20 objectives. Objectives used to capture images include Nikon achroplan x10 and x60. Fluorescent images were acquired using a 60x, 1.45NA achroplan objective, and a digital camera controlled by metamorph software. Differential interference contrast (DIC) images were acquired using a Zeiss Axiovert 200 M inverted time-lapse microscope equipped with a 37°C incubation chamber and motorised stage. All Images were acquired every 5 minutes for up to 24 hours. Peripheral devices were controlled using Metamorph software (Molecular Devices) on the Nikon microscope and Andor iQ software (Andor Technology) on the Zeiss microscope.

### Cell tracking and quantitative data analysis

The Manual Tracking plugin for ImageJ was used to track cells remaining within the overlapping fields of view created by the time-lapse images. The chemotaxis plugin was used to analyse cell behaviour. MATLAB was used to examine the data, produce the various plots and apply the Rayleigh test.

## Supporting Information

Movie S1 **Control experiment.** MV3 cells migrating over the narrow bridge of the Insall chamber in the presence of SFM only. The images were taken every 5 minutes for 12 hours using a 10x phase objective.
(MOV)

Movie S2 **Chemotaxis experiment.** MV3 cells migrating over the narrow bridge of the Insall chamber in the presence of a 10% FBS gradient to the right. The images were taken every 5 minutes for 12 hours using a 10x phase objective.
(MOV)
